# Star Sensor Denoising Algorithm Based on Edge Protection

**DOI:** 10.3390/s21165255

**Published:** 2021-08-04

**Authors:** Kaili Lu, Enhai Liu, Rujin Zhao, Hui Zhang, Hong Tian

**Affiliations:** 1 Institute of Optics and Electronics, Chinese Academy of Sciences, Chengdu 610000, China; lukaili@ioe.ac.cn (K.L.); zhaorj@ioe.ac.cn (R.Z.); hzhang@ioe.ac.cn (H.Z.); tianhong@ioe.ac.cn (H.T.); 2 Key Laboratory of Science and Technology on Space Optoelectronic Precision Measurement, Chinese Academy of Sciences, Chengdu 610000, China

**Keywords:** single-pixel noise, star sensor, denoising, subtemplates, reconstruction function

## Abstract

Single-pixel noise commonly appearing in a star sensor can cause an unexpected error in centroid extraction. To overcome this problem, this paper proposes a star image denoising algorithm, named Improved Gaussian Side Window Filtering (IGSWF). Firstly, the IGSWF algorithm uses four special triangular Gaussian subtemplates for edge protection. Secondly, it exploits a reconstruction function based on the characteristic of stars and noise. The proposed IGSWF algorithm was successfully verified through simulations and evaluated in a star sensor. The experimental results indicated that the IGSWF algorithm performed better in preserving the shape of stars and eliminating the single-pixel noise and the centroid estimation error (CEE) value after using the IGSWF algorithm was eight times smaller than the original value, six times smaller than that after traditional window filtering, and three times smaller than that after the side window filtering.

## 1. Introduction

A star sensor [1] is a high-precision attitude measurement instrument that takes a star as a working object and calculates multiple reference vectors using multiple stars. The centroid extraction [2,3] of the star-point region has a great influence on the final precision of a star sensor. The factor affecting accuracy of centroid extraction can be attributed to the motion blur and the background noise [4]. The motion blur is generated by the fast motion of satellite under dynamic conditions. It will bring about the blur of the stars. There have been many studies [5,6,7,8] on this aspect. Most of them focused on the method of restoration of the star image. Generally, the background noise can be divided into two categories: single-pixel noise with only a single pixel, and large-area noise with a continuous change in the gray level. Single-pixel noise can be attributed to two main sources: the nonuniform response of a complementary metal oxide semiconductor (CMOS) detector [9] and the impact of cosmic radiation particles [10]. Large-area noise with a continuous change in the gray level is usually influenced by sunlight, moonlight, and earth-atmosphere light [11]. Considering the actual engineering requirements in the aerospace field, this paper aimed to develop a single-point noise elimination method.

**Figure 1:**
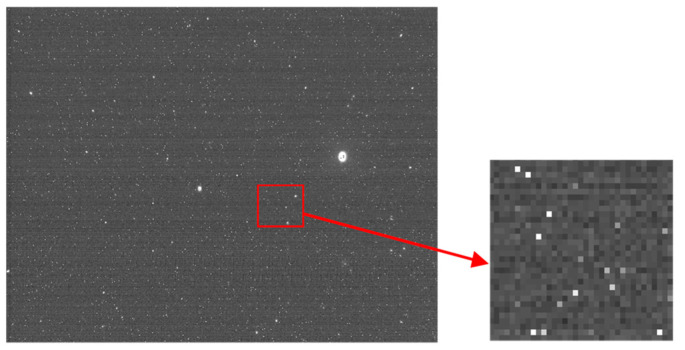
The noise caused by the impact of cosmic radiation particles.

**Figure 2:**
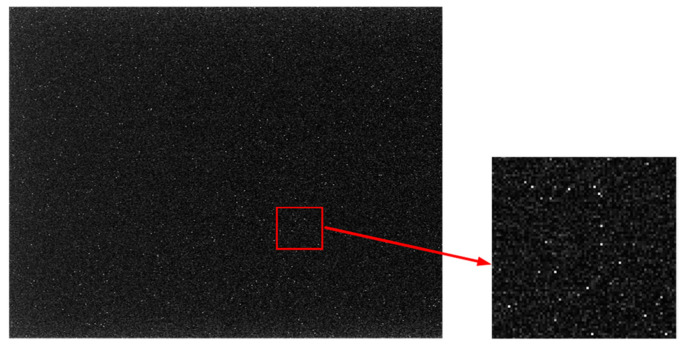
The noise caused by a nonuniform response of a CMOS detector.

As shown in Figure 1 and Figure 2, the nonuniform response of a CMOS detector and the cosmic radiation particles can cause significant dense single-pixel noise. To deal with the single-pixel noise, traditional window filter algorithms, such as BOX filtering [12], mean filtering [13], and Gaussian filtering [14,15], can be used. However, these methods have a kinetic disadvantage, and the original image can be damaged during the denoising process. This disadvantage will be discussed in detail in Section 2, and the traditional window filter algorithm will be compared with the proposed Improved Gaussian Side Window Filtering (IGSWF) algorithm in Section 3.

Recently, many studies on single-pixel noise and star-sensor applications have been conducted. Schmidt [16] proposed a method to deal with the single-pixel noise by using the background value prediction, where the background value is obtained through multiframe accumulation and the noise is eliminated using subtraction. However, this method places high requirements on the speed of the star sensor, and it is suitable only for the nonuniform response of a CMOS detector. Zheng [17] proposed an improved method based on the Schmidt method and adjusted the scale of the correction domain at high speed, which could adapt to the higher dynamic speed. The results showed that this method could merely deal with the nonuniform response of the CMOS detector having a fixed position, but random noise caused by cosmic radiation particles could not be handled well. The two above-mentioned algorithms cannot eliminate the two types of single-pixel noise at the same time without destroying the precision of a star sensor. Hence, the methods for eliminating single-pixel noise still face challenges, and further analyses are necessary.

To deal with the single-pixel noise caused by both a nonuniform response of a CMOS detector and cosmic radiation particles simultaneously, this paper proposes a denoising algorithm, named IGSWF, which is based on edge protection.

The proposed algorithm is based on the side window filtering (SWF) algorithm proposed by Hui Yin [18], which can smooth the background noise and prevent the object boundary from being damaged at the same time. In addition, the algorithm is improved for application in star images. Firstly, unlike SWF, the proposed IGSWF algorithm uses four triangular Gaussian subtemplates for noise filtering. Secondly, based on the shape and energy characteristics of the star point, background, and image noise, a suitable calculation function $f_{min}\;$ for eliminating single-pixel noise in a star image was defined. The proposed algorithm was verified by experiments, and the experimental results showed that the proposed IGSWF algorithm can effectively protect the edge of the preprocessed star point from being damaged and successfully eliminate the single-pixel noise in a star image at the same time. Finally, it was verified that the proposed IGSWF algorithm was favorable to improving the precision of centroid extraction and the accuracy of star identification.

The remainder of this paper is organized as follows: Section 2 explains the SWF principles and introduces the proposed IGSWF algorithm. Section 3 presents the experimental results. Finally, Section 4 and Section 5 conclude the paper and present future directions for work.

## 2. Materials and Methods

### 2.1. Side Window Filtering Principle

In this section, the principle of side window filtering (SWF) is introduced.

The coordinates $\left(x_{0},y_{0}\right)\;$ of the center of mass can be expressed in the following way:

(1) $$\left\{ \begin{array}{c} x_{0}=\frac{\sum_{x=1}^{m}\sum_{y=1}^{n}(F_{s_{c}}\left(x,y\right)+F_{s_{e}}\left(x,y\right)+F_{b}\left(x,y\right))x}{\sum_{x=1}^{m}\sum_{y=1}^{n}(F_{s_{c}}\left(x,y\right)+F_{s_{e}}\left(x,y\right)+F_{b}\left(x,y\right))}\\ y_{0}=\frac{\sum_{x=1}^{m}\sum_{y=1}^{n}(F_{s_{c}}\left(x,y\right)+F_{s_{e}}\left(x,y\right)+F_{b}\left(x,y\right))y}{\sum_{x=1}^{m}\sum_{y=1}^{n}(F_{s_{c}}\left(x,y\right)+F_{s_{e}}\left(x,y\right)+F_{b}\left(x,y\right))} \end{array},\right.$$

where $F_{s_{c}}\left(x,y\right)$ denotes the central pixel of a star, $F_{s_{e}}\left(x,y\right)$ denotes the pixel on the star edge, and $F_{b}\left(x,y\right)$ represents the background.

In the field of visual processing, window-smoothing filtering is a common method for denoising, and some of the most common filtering methods are box filtering, mean filtering, and Gaussian filtering. Window-smoothing filtering can be expressed as:

(2) $$I_{o}=\underset{\omega_{j}\in B}{\sum }\omega_{j}I_{j},\;$$

where B represents the filtering window centered at the current pixel j,$\;I_{j}$ is the input image region, $I_{o}$ is the output pixel, and $\omega_{j}$ denotes the weight value.

**Figure 3:**
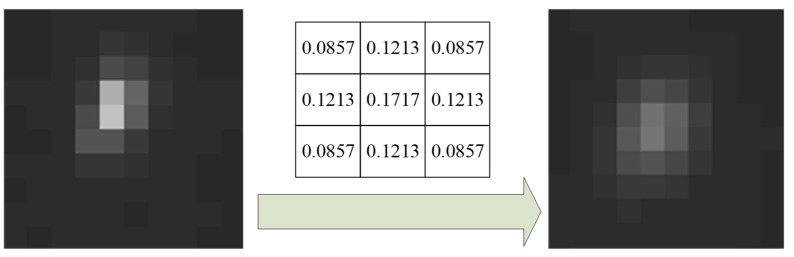
The result of traditional Gaussian filtering.

To demonstrate the filtering effect, traditional Gaussian filtering with a window size *r* of 3 and a scale σ of 0.8 was applied to a star image, and the results are presented in Figure 3.

As shown in Figure 3, after denoising by Gaussian filtering, the inherent shape of the star point was destroyed, the energy of the star point was dispersed, and the edge became blurred. Thus, there was a significant drop in the values of $F_{s_{c}}\left(x,y\right)$ and $F_{s_{e}}\left(x,y\right)$ in Equation (1). Accordingly, the weight values of the central pixel and the pixel on the star edge decreased, which resulted in a deviation of ($x_{0},y_{0}$). Hence, a method to preserve the energy and edge of the star point is urgently needed.

Furthermore, Taylor’s expansion [19] of a pixel (x,y) can be expressed as:

(3) $$f\left(x,y\right)=f\left(x_{0},y\right)+\frac{f^{\prime }\left(x_{0},y\right)}{1!}\left(x-x_{0}\right)+\frac{f^{\prime \prime }\left(x_{0},y\right)}{2!}{\left(x-x_{0}\right)}^{2}+\ldots ,$$

where f(x,y) denotes the intensity value at the pixel (x,y).

For an edge point  (x,y), the left limit (x−ε,y) and the right limit (x+ε,y) can be defined when ε>0.

Assuming that $x_{0}=x-\varepsilon$ and function f(x,y) is differentiable at $x_{0}$, then Taylor’s expansion of a pixel (x,y) can be expressed as:

(4) $$f\left(x-2\varepsilon ,y\right)\approx f\left(x_{0}\right)+f^{\prime }\left(x_{0}\right)\left(x-x_{0}\right)=f\left(x-\varepsilon ,y\right)+f^{\prime \left(x-\varepsilon ,y\right)}\left(-\varepsilon \right).$$

Similarly, when $x_{0}=x+\varepsilon$, Taylor’s expansion of a pixel (x,y) becomes:

(5) $$f\left(x+2\varepsilon ,y\right)\approx f\left(x_{0}\right)+f^{\prime }\left(x_{0}\right)\left(x-x_{0}\right)=f\left(x+\varepsilon ,y\right)+f^{\prime \left(x+\varepsilon ,y\right)}\left(\varepsilon \right).$$

According to Equations (4) and (5), the pixel value on one side of the edge point can be obtained by the pixel value in the field on that side. Therefore, it is necessary to ensure that the boundary cannot be crossed during the filtering process. In other words, when a pixel j is positioned on the image edge, the edge of a filter B should be aligned with the center pixel j. Therefore, the SWF algorithm takes each pixel as a potential edge point and generates several different filtering subwindows. Then, it aligns the edge or a corner position of the filtering window with the edge point. Finally, the best reconstruction result after filtering is selected as the final filtering result.

### 2.2. The Proposed Improved Gaussian Side Window Filtering Algorithm

As mentioned before, the proposed IGSWF algorithm is based on SWF. The main objective of IGSWF is to remove the single-pixel noise from a star image while ensuring that the shape and energy characteristics of the stars are not affected.

The main innovation of the proposed IGSWF algorithm was that four triangular Gaussian subtemplates were designed, and a suitable reconstruction function $f_{min}$ was defined to obtain the best-possible final filtering results. The proposed IGSWF was more suitable than the SWF for star image denoising.

#### 2.2.1. Filter Template Design

According to the description in Section 2.1, a series of filter templates should be designed when the current processing pixel is aligned with the edge or corner position of a filter subtemplate. Considering that the distribution of star points on a detector is similar to the Gaussian distribution, the Gaussian filter template was chosen as the final filter template in this work.

**Figure 4:**
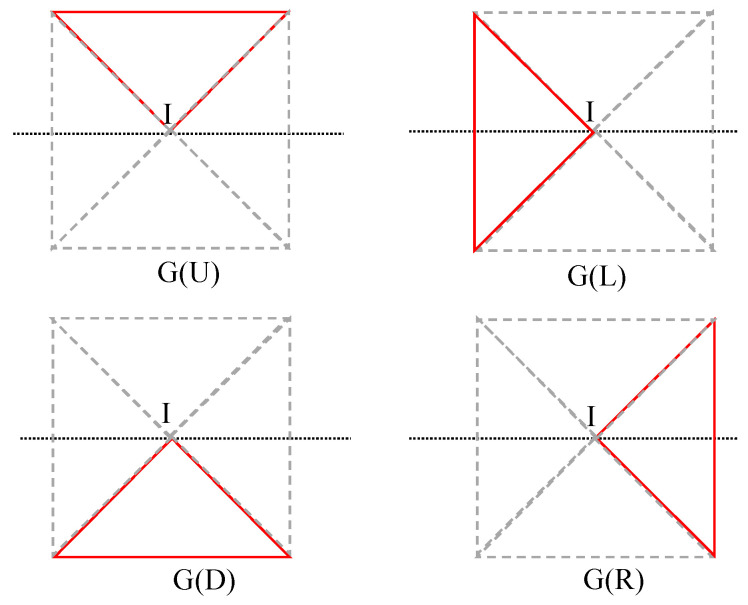
The four triangular Gaussian subtemplates.

Aiming at a full consideration of the edge feature and processing capacity of a processing chip (which is usually a field programmable gate array) at the same time, the traditional Gaussian filter template is separated into four subtemplates, namely, up, down, left, and right subtemplates, as shown in Figure 4. The shape of the subtemplates is the same (a triangle). The four filtering templates are used simultaneously during the filtering process.

#### 2.2.2. Improved Gaussian Side Window Filtering Steps

**Figure 5:**
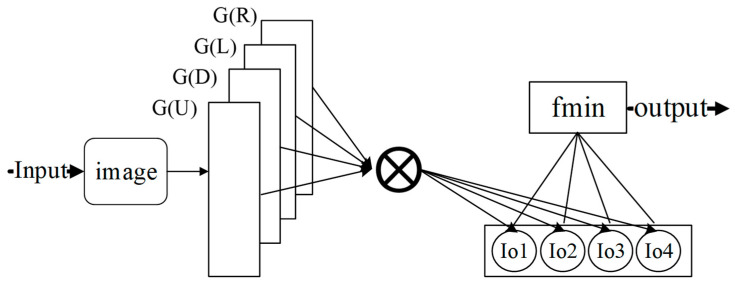
The block diagram of the IGSWF algorithm.

The block diagram of the IGSWF algorithm is presented in Figure 5, where it can be seen that it includes three main steps: Gaussian subtemplate convolution, calculation of relative minimum of filtering values, and the final image determination.

Step 1: Gaussian Subtemplate Convolution

The pixels are convolved using the Gaussian subtemplates in the four directions: up, down, left, and right. Based on Equation (2), the four following expressions can be obtained as follows:

(6) $$I_{o2}=\underset{\left(i,j\right)\in G\left(L\right)}{\sum }\omega_{\left(i,j\right)}I_{\left(i,j\right)},\;I_{o3}=\underset{\left(i,j\right)\in G\left(D\right)}{\sum }\omega_{\left(i,j\right)}I_{\left(i,j\right)},\;I_{o4}=\underset{\left(i,j\right)\in G\left(R\right)}{\sum }\omega_{\left(i,j\right)}I_{\left(i,j\right)},\;\mathrm{and}\;I_{o1}=\underset{\left(i,j\right)\in G\left(U\right)}{\sum }\omega_{\left(i,j\right)}I_{\left(i,j\right)},$$

where $I_{o1},I_{o2},I_{o3},$ and $I_{o4}\;$ represent the filtering values of the four templates in the up, left, down, and right directions, respectively.

Step 2: Relative Minimum of the Filtering Values

The relative minimum of the filtering values of the four templates is expressed as follows:

(7) $$I_{m}=f_{min}\left(I_{o1},I_{o2},I_{o3},I_{o4},I_{c}\right),$$

where $I_{m}$ represents the relative minimum value; and $I_{c}\;$ represents the input value, which is also the center pixel. The main objective is to find the best reconstruction function $f_{min}$, which will be discussed in detail in Section 2.2.3.

Step 3: Final Image Determination

**Figure 6:**
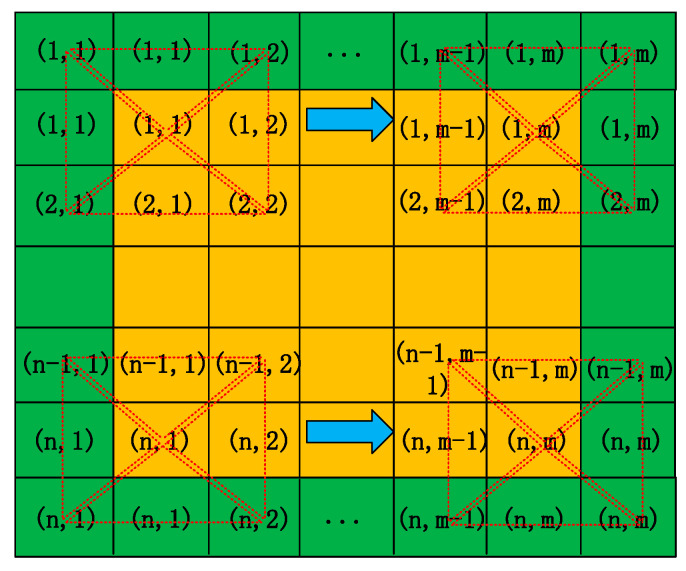
Filter template processing.

The output image after denoising is generated based on the calculated pixel instead of the original pixel. Step 1 is repeated until the last pixel has been calculated. As shown in Figure 6, the surrounding edge of the image (green fields) is filled with adjacent pixels (yellow fields) uniformly, and the filter templates (red dotted line) shift from left to right and from top to bottom.

#### 2.2.3. Reconstruction Function Calculation

**Figure 7:**
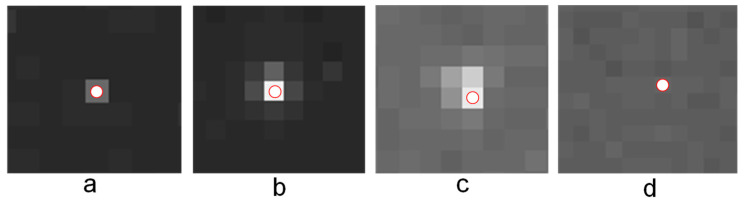
Four types of pixels in a star image: (**a**) a single pixel, which is characterized by a relatively bright pixel in the center and flat black pixels around it; (**b**) a standard central pixel of a star point, which is characterized by a relatively bright center pixel and approximate Gaussian distribution of the surrounding pixels; (**c**) an irregular central pixel of the Gaussian star point, which is characterized by a relatively bright pixel in the center and approximate Gaussian distribution on several sides, and the remaining part is the gentle background; and (**d**) pixels in a flat region with a small difference from the other pixels.

The calculation of the reconstruction function $f_{min}$ is mainly based on the shape and energy characteristics of the star point, background, and image noise. Therefore, by analyzing a large amount of shooting data, pixels in a star image can be categorized into four types, as shown in Figure 7.

According to the classification in Figure 7, the reconstruction function $f_{min}$, which is suitable for a star image, can be defined as follows.

For the type presented in Figure 7a, the reconstruction function $f_{min}$ satisfies condition A, which is as follows:

(8) $$A=\{I_{ok}<I_{c}\;\&\;\Delta I_{c}<T_{dmin}\;\&\;(I_{c}-I_{min})>T_{dmin}\}$$

For the type presented in Figure 7b, the reconstruction function $f_{min}$ satisfies condition B, which is as follows:

(9) $$B=\left\{I_{ok}<I_{c}\;\&\;\left((I_{c}-I_{min}\right)>T_{dmax}\;|\;\left(I_{max}-I_{min}\right)>T_{dela}\right\}$$

For the types presented in Figure 7c,d, this means that the remaining pixels do not satisfy conditions A and B at the same time.

In the above-mentioned condition, $I_{ok}$ (*k* = 1:4) represents the filtering value of a subtemplate in the up, left, down, or right direction; $I_{c}$ represents the current pixel; $\Delta I_{c}$ represents the d-value around the current pixel $I_{c}$; $I_{max}$ represents the maximum $L_{2}$ distance; $I_{min}$ represents the minimum $L_{2}$ distance; $T_{dmin}$ is the lower limit for the d-value of smooth background; $T_{dmax}$ is the lower limit for the d-value of $I_{c}$ and $I_{min}$; and lastly, $T_{dela}$ is the lower limit for the d-value of $I_{max}$ and $I_{min}$.

The maximum $L_{2}$ distance $I_{max}$, the minimum $L_{2}$ distance $I_{min}$, and the d-value $\Delta I_{c}$ are calculated as follows:

(10) $$I_{max}=argmax_{k\epsilon \left\{1,2,3,4\right\}}{\left||I_{ok}-I_{c}|\right|}_{2}^{2}\;I_{min}=argmin_{k\epsilon \left\{1,2,3,4\right\}}{\left||I_{ok}-I_{c}|\right|}_{2}^{2}\;\mathrm{and}\;\Delta I_{c}=\{\begin{array}{c} \begin{array}{c} I_{c}\left(i-1,j\right)-I_{c}\left(i-2,j\right)\\ I_{c}\left(i+1,j\right)-I_{c}\left(i+2,j\right)\\ I_{c}\left(i,j-1\right)-I_{c}\left(i,j-2\right) \end{array}\\ I_{c}\left(i,j+1\right)-I_{c}\left(i,j+2\right). \end{array}$$

In addition, several constants, namely, $T_{dmin}$, $T_{dmax}$, and $T_{dela}$ are set as follows:

(11) $$T_{dmin}=8;T_{dmax}=65;T_{dela}=20.$$

The above three constants are related to the gray distribution of the background and star. They are chosen according to empirical values, and they have been tested using different scenes. The effect of IGSWF on noise was also verified by the experiment, as presented below.

When a pixel is under condition A, it represents single-pixel noise. As mentioned before, single-pixel noise should be eliminated. Therefore, it is necessary to maximize the distance between the input and output pixels, which means that the output pixel obtained after filtering should be as far as possible from the input pixel and as close as possible to the background pixel. In this work, the maximum $L_{2}$ distance denoted as $I_{max}$ was calculated and multiplied by 0.9 (it represented an approximation coefficient used to make the result closer to the background than $I_{max}$); in this way, the noise is weakened to bring it to the level of the background.

When a pixel is under condition B, it represents the brightest pixel; to maintain the star centroid, the output value is set to be the same as the input.

When a pixel is under other conditions, it mainly represents the star edges and background. To preserve both edges and energy, it is necessary to minimize the distance between the input and output pixels, which means that the output pixel obtained after filtering should be as close as possible to the input pixel. Thus, the minimum $L_{2}$ distance $I_{min}$ is chosen as the output value in this case.

According to the characteristic given above, function $f_{min}$ can be obtained as:

(12) $$f_{min}=\{\begin{array}{c} 0.9*I_{max}\;\;\;\;\;\;\;\;\;\;\;\;\;\;condition\;A\\ I_{c}\;\;\;\;\;\;\;\;\;\;\;\;\;\;\;\;\;\;\;condition\;B\\ I_{min}\;\;\;\;\;\;\;\;\;\;\;\;\;\;\;\;\;\;\;\;\;other \end{array}.$$

By using function $f_{min}$, the optimum output value can be obtained, which can replace the input value.

#### 2.2.4. Filter Window Parameter Selection

As shown in Figure 4, the collection S can be obtained as:

(13) $$S=\left\{G\left(U\right),G\left(L\right),G\left(D\right),G\left(R\right)\right\}=\mathrm{guass}\left(r,\sigma \right)=\frac{1}{2\pi \sigma }e^{-({\left(x-\frac{r-1}{2}-1\right)}^{2}+\left(y-\frac{r-1}{2}-1{)}^{2}\right)/2\sigma^{2}}$$

In other words, the four subtemplates can be combined into a two-dimensional Gaussian template, and thus the next key point is to select proper values of the Gaussian window size r and scale σ.

The size of the star points on the focal plane of a CMOS detector is usually between 2 × 2 and 7 × 7. Hence, the range of the Gaussian window size should be from 3 × 3 to 7 × 7.

Because single-pixel noise should be eliminated as much as possible, σ should be as large as possible. However, a larger σ will weaken the energy distribution of a star, and appropriate values of σ were found to be 0.8, 1.0, and 1.2.

**Figure 8:**
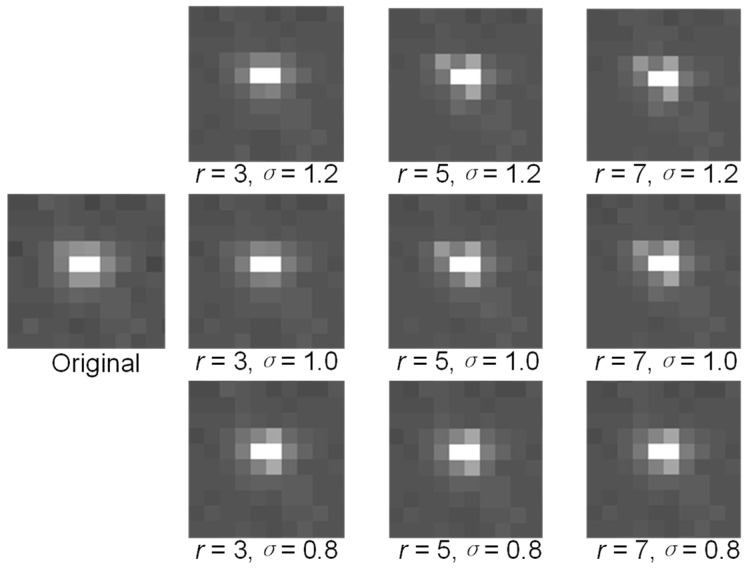
Results for different values of r and σ.

As shown in Figure 8, at the same value of σ, the increase in the filtering window size r results in an abnormal energy distribution of star points. Considering that the high-brightness area is usually small and the Gaussian window size r is larger, the star edge can be destroyed, as shown in Figure 8, where  r = 5 and r = 7.

Therefore, when the Gaussian window size r was set to 3 and the scale σ was set to 1.2, the ideal original shape of the star point could be obtained, and the noise could be suppressed to a greater extent.

## 3. Results

### 3.1. Experimental Conditions

In this section, the performance of the proposed IGSWF algorithm in protecting the shape of stars and eliminating the single-pixel noise was evaluated experimentally.

Firstly, a simulation experiment was carried out. An actual star image with a resolution of 1536 × 1536 that was taken by a certain type of star sensor was used in the experiment. The operating platform was a 2.5 GHz Intel I7 CPU with 16 GB of memory, and the simulation software was MATLAB R2012b. The proposed IGSWF algorithm was used to substantiate the effect in eliminating single-pixel noise. It was also compared with Gaussian filtering, box filtering, mean filtering, and SWF in preserving the shape of a star point during the denoising process.

Secondly, an application experiment was carried out. The IGSWF algorithm was applied to a star sensor when it docked with the dynamical star simulator. The star sensor was made using the AM3358 processor and the CMV4000 detector. The resolution of the detector in the star sensor was 1536 × 1536, and the field of view was 15°×15°. The star points in the dynamic star simulator moved at a rate of 0.06°/s, which is the normal operating rate of a satellite. The programming IDE tool was CCS v5.4. The centroid estimation error (CEE) [20] for six kinds of stars with different sizes was analyzed. In addition, the proposed IGSWF algorithm was compared with Gaussian filtering, box filtering, mean filtering, Zheng processing [17], and SWF in the value of the centroid estimation error (CEE). The accuracy of star identification [21] was also tested when we chose different algorithms.

**Figure 9:**
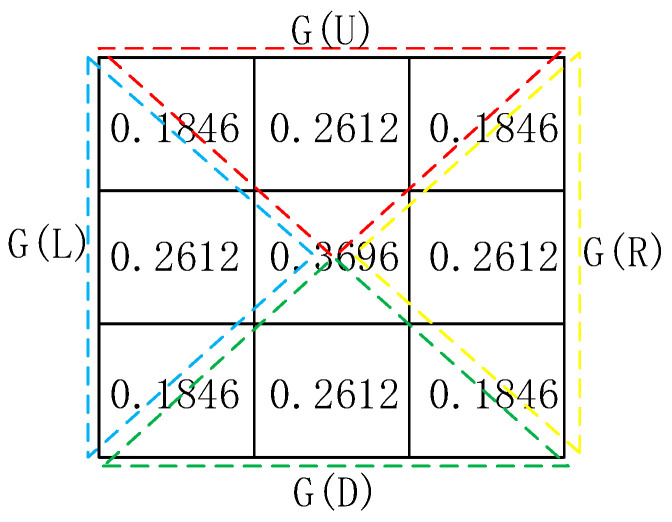
Final filtering parameters.

The specific coefficients of the four subtemplates are shown in Figure 9. The values of these coefficients could be obtained from Equation (13). The coefficients were guaranteed to meet the normalization conditions.

### 3.2. Simulation Experiment

#### 3.2.1. Denoising Effect on the Star Image

**Figure 10:**
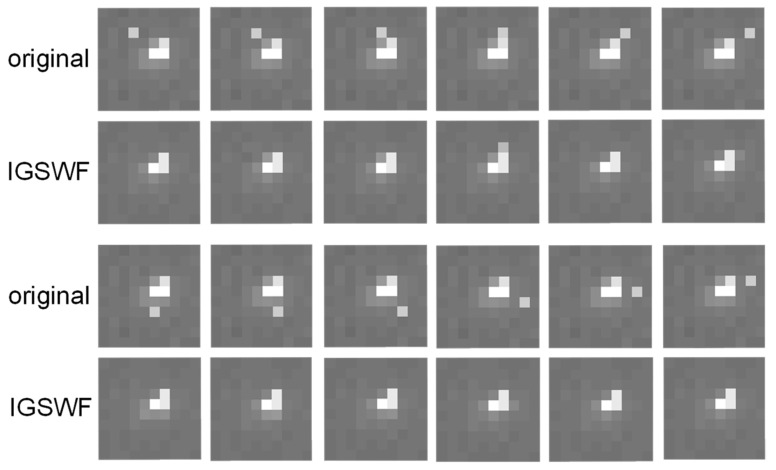
Star-point region before and after denoising.

Firstly, the denoising effect of IGSWF on a star image with single-pixel noise was analyzed. As shown in Figure 10, the single-pixel noise position in the star image was not fixed in the original image. The local star-point region before and after IGSWF denoising was compared.

The results in Figure 10 indicated that wherever the single-pixel noise existed in a star image, the IGSWF algorithm could effectively filter the noise without destroying the star shape. Therefore, by applying the proposed algorithm, the star-point region could be kept smooth while mitigating the influence on the star shape, which was beneficial for the calculation of the center of mass in the star-point region.

**Figure 11:**
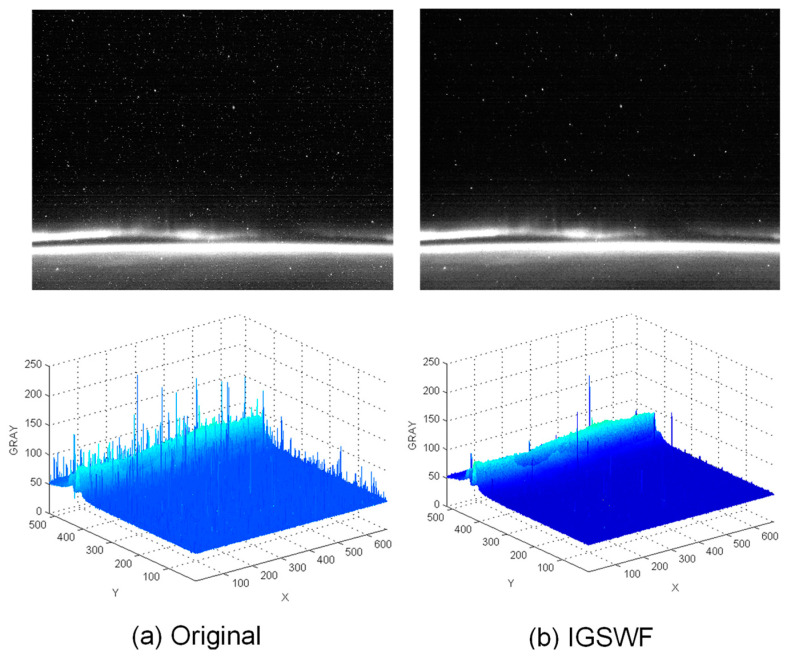
Comparison before and after denoising in SAA area 1: (**a**) the star image and the three-dimensional distribution of the grayscale before denoising; (**b**) the star image and the three-dimensional distribution of the grayscale after IGSWF processing.

**Figure 12:**
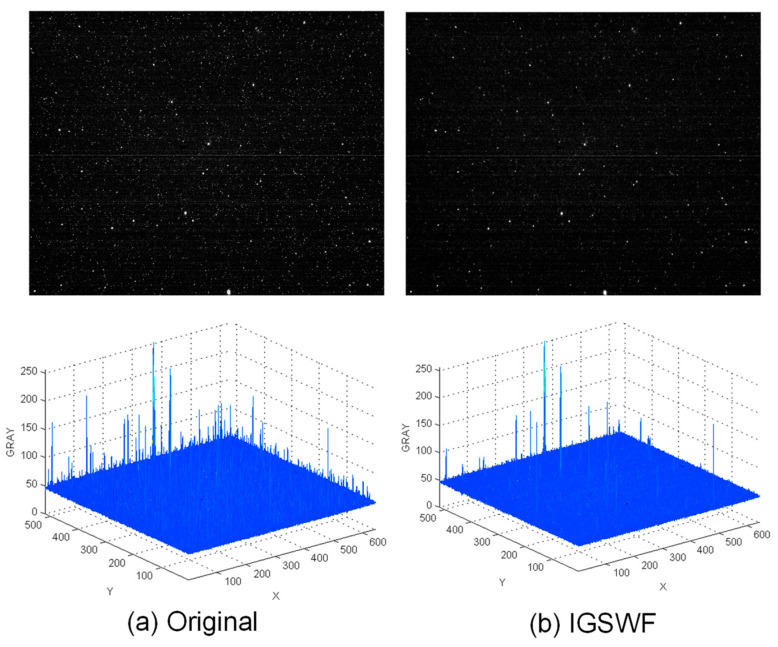
Comparison before and after denoising in SAA area 2: (**a**) the star image and the three-dimensional distribution of the grayscale before denoising; (**b**) the star image and the three-dimensional distribution of the grayscale after IGSWF processing.

Secondly, as shown in Figure 11 and Figure 12, the actual star image, which was taken in orbit by a certain type of star sensor, was simulated.

When a star sensor is in strong radiation zones, such as the South Atlantic Anomaly (SAA area) [22], there will be much random single-pixel noise in a star image, while fixed single-pixel noise is also generated when a star sensor is running at a high temperature. Therefore, the real effect in the original and denoised images was compared. The three-dimensional distribution of the grayscale in the star image was also used to analyze the performance of IGSWF.

The results shown in Figure 11 and Figure 12 indicate that the star image became much cleaner and smoother after IGSWF processing regardless of the star sensor’s position. Moreover, the denoising effect of IGSWF was not affected by the earth-atmosphere light. Furthermore, a large amount of single-pixel noise encountered in orbit was effectively suppressed by IGSWF, while the gray level of the star point remained unchanged.

Therefore, based on the results, it can be concluded that the IGSWF algorithm is suitable for the application in orbit and can deal with a severe single-pixel noise problem in the SAA area. The IGSWF algorithm can be extended to other scenarios that include radiation.

#### 3.2.2. Comparison with Other Algorithms

The performance of IGSWF was compared with those of the traditional Gaussian filter, box filter, and mean filter for the case of a star image with the single-pixel noise. The star image, the three-dimensional distribution of the grayscale, and the local star-point region were compared for different algorithms.

**Figure 13:**
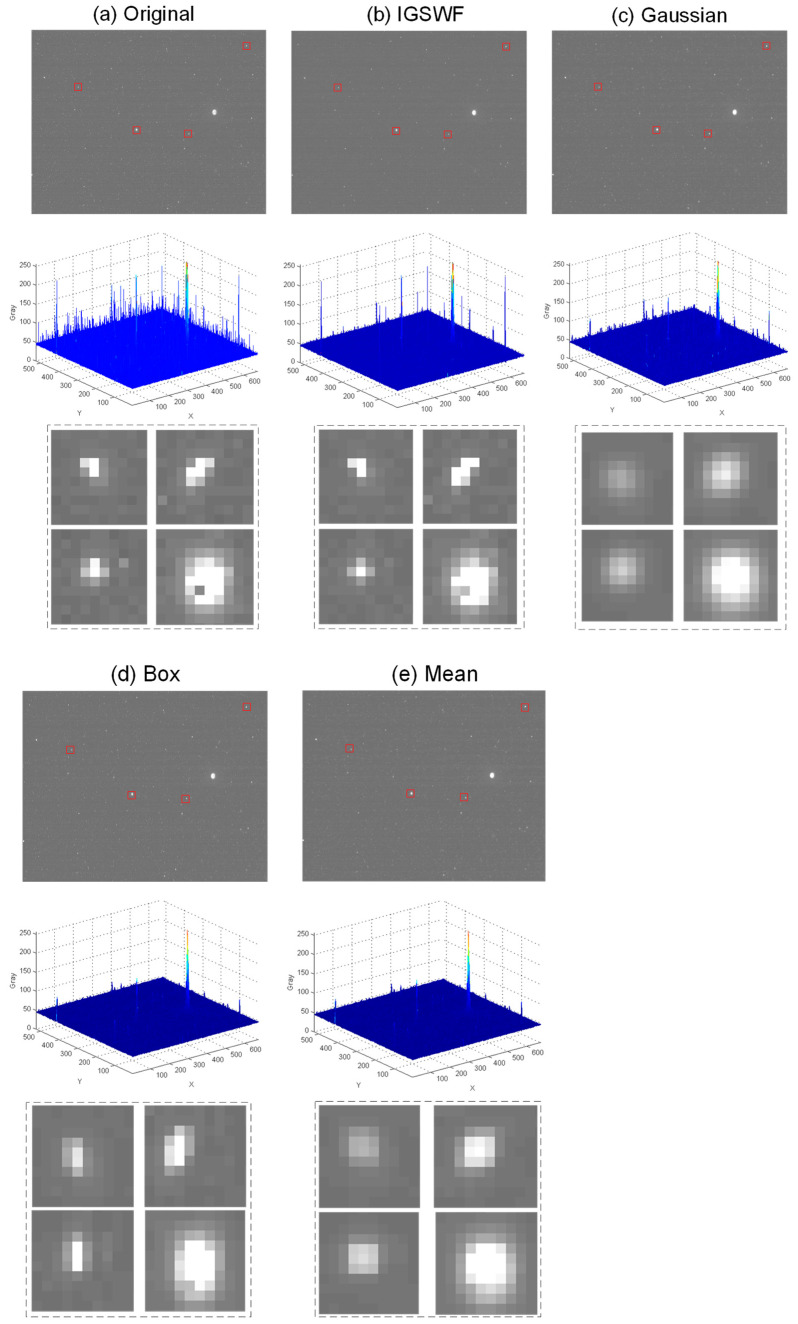
Comparison of the IGSWF algorithm with Gaussian filtering, box filtering, and mean filtering: (**a**) before denoising; (**b**–**e**) after denoising by the IGSWF algorithm, Gaussian filtering, box filtering, and mean filtering, respectively. The first lines are star images, the second lines are the three-dimensional distribution of the grayscale in the star image, and the third lines are the local star points.

From Figure 13a–e, it can be found that the star image after the IGSWF algorithm was cleaner than the original image. Compared with the other algorithms, the background area after IGSWF was smoother. In addition, the gray value of the star after IGSWF changed less than after the other filtering algorithms, and the gray value of the single-pixel noise was practically invisible. In addition, the IGSWF algorithm could keep the original shape and energy distribution of the star points relatively unchanged while denoising. This advantage was not observed in the traditional algorithms. Therefore, the IGSWF algorithm had a stronger ability to protect the star shape and eliminate the single-pixel noise from a star image than the traditional Gaussian filtering, box filtering, and mean filtering algorithms.

**Figure 14:**
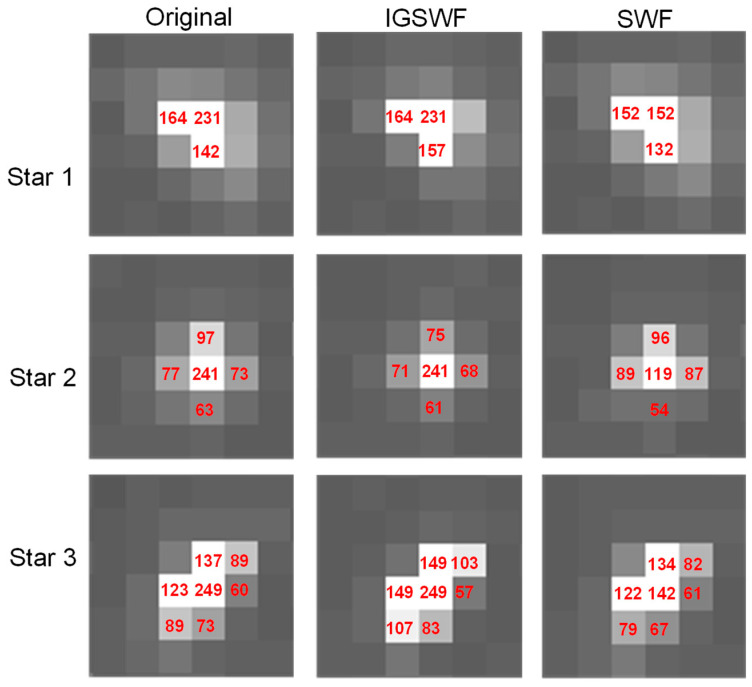
Comparison between IGSWF and SWF.

At the same time, the proposed IGSWF was compared with SWF regarding energy distribution and precision. Because SWF had a similar effect in protecting the star shape as the IGSWF algorithm, only the differences in the energy distribution and precision of star points were analyzed. In Figure 14, the first column represents the original energy distribution, the second column represents the energy distribution after IGSWF, and the third column represents the energy distribution after SWF. The relative proportion of the grayscale in the original star image was the closest to that after the IGSWF, which indicated that IGSWF maintained the energy distribution better than the SWF.

In Figure 14, the center of mass was calculated, and the calculation results are shown in Table 1.

Then, the deviation between the different centers of mass was calculated by:

(14) $$\varepsilon_{n}=\sqrt{{\left(x_{n}-x_{o}\right)}^{2}+{\left(y_{n}-y_{o}\right)}^{2}}\;\mathrm{and}\;{\varepsilon^{\prime }}_{n}=\sqrt{{\left({x^{\prime }}_{n}-x_{o}\right)}^{2}+{\left({y^{\prime }}_{n}-y_{o}\right)}^{2}},$$

and it was obtained that:

(15) $$\varepsilon_{1}=\;0.036249,\;\varepsilon_{2}=\;0.044777,\varepsilon_{3}=0.030529\;\mathrm{and}\;{\varepsilon^{\prime }}_{1}=0.091093,{\varepsilon^{\prime }}_{2}=\;0.101139,{\varepsilon^{\prime }}_{3}=0.068797$$

By comparing $\varepsilon_{n}$ with ${\varepsilon^{\prime }}_{n}$, it was found that the center of mass after IGSWF was closer to the original center of mass than that after the SWF. Consequently, the proposed IGSWF algorithm can protect the energy distribution better than the SWF, and IGSWF is more favorable for centroid extraction than SWF.

### 3.3. Application Experiment

**Figure 15:**
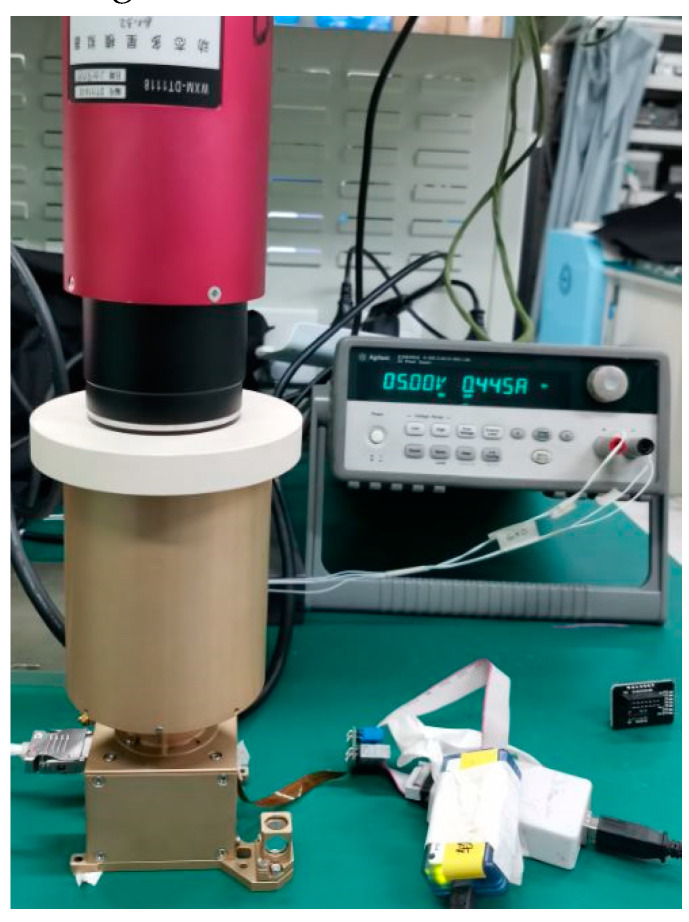
The verification platform.

**Figure 16:**
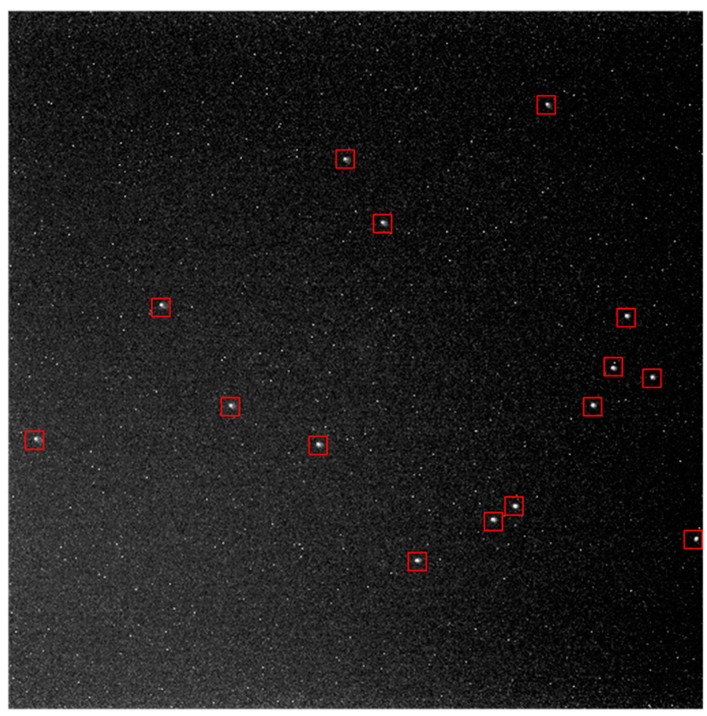
Star image created by the dynamic star simulator.

In Figure 15 and Figure 16, the dynamic star simulator was used to generate star points, and the star sensor was aligned with the optical center of the dynamic star simulator for testing.

To show the accuracy of centroid extraction, the CEE value was introduced. The CEE value represented the variance between the theoretical and actual centers of mass, and it was calculated by:

(16) $$\mathrm{CEE}=\frac{1}{n}\overset{n}{\underset{i=1}{\sum }}\sqrt{{\left(x_{i}-x_{c}\right)}^{2}+{\left(y_{i}-y_{c}\right)}^{2}}$$

where *n* denoted the actual number of available star points obtained in each frame of the star image, ($x_{i},y_{i}$) was the centroid position of the *i*th star point calculated in the current frame image, and ($x_{c},y_{c}$) represented the theoretical centroid position of the *i*th star point in the current frame image. The theoretical centroid position was obtained using the star simulator. In the meantime, the centroid extraction with the threshold was used as a centroid extraction method.

**Figure 17:**
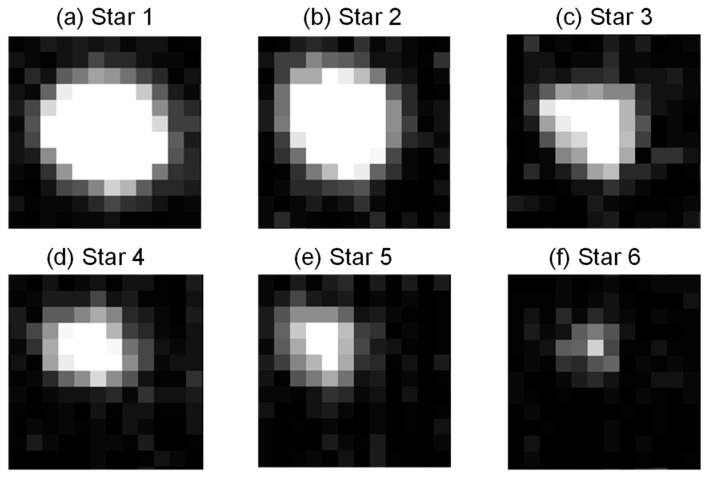
The six kinds of stars with different sizes: (**a**–**f**) Star 1 to Star 6 corresponded to 1 Mv, 2 Mv, 3 Mv, 4 Mv, 5 Mv, and 6 Mv, respectively.

Firstly, in Figure 17, the dynamic star simulator generated six kinds of stars with different sizes. The six kinds of sizes corresponded to six kinds of star magnitude. Star 1 to Star 6 corresponded to 1 Mv, 2 Mv, 3 Mv, 4 Mv, 5 Mv, and 6 Mv, respectively. We calculated the CEE value (*n* = 1) for each star after IGSWF processing when the stars were rolling.

**Figure 18:**
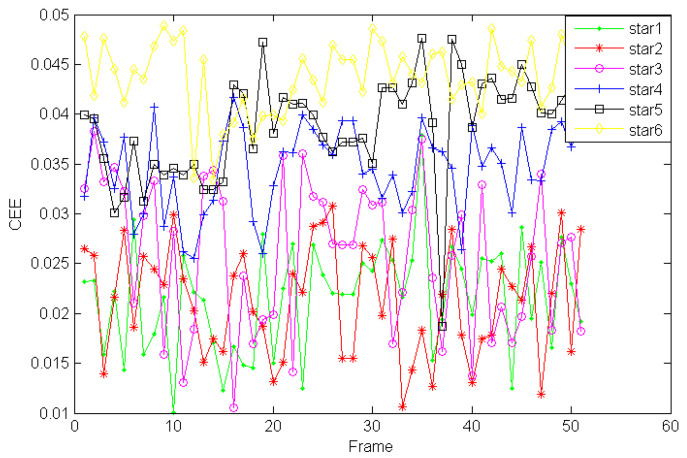
The CEE curves of the different stars.

As shown in Figure 18, it could be found that the CEE value was smaller if the size of the star was larger. This means that the center of mass of a star with larger size was much closer to original state after IGSWF filtering.

Secondly, in this experiment, the performance of centroid extraction was evaluated for the star image in the original state and the star image after Gaussian filtering, box filtering, mean filtering, Zheng processing [17], SWF processing, and IGSWF processing. The CEE curves for 1000 continuous frames of images were obtained, and the error curves before and after correction were drawn.

**Figure 19:**
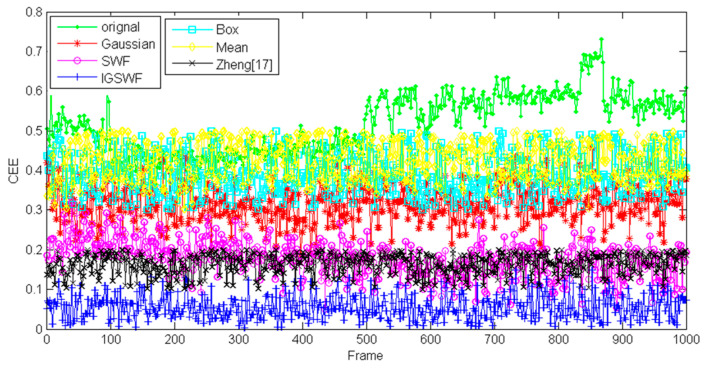
Comparison of the CEE curves of the different algorithms.

As shown in Figure 19, the deviation of the centroid extraction result was very large before denoising, and the CEE value fluctuated around 0.5. When traditional Gaussian filtering was adopted, the CEE value was superior to the original value and fluctuated around 0.35. When box filtering or mean filtering was adopted, the CEE value fluctuated around 0.4. After denoising by the SWF method, the deviation of the centroid extraction result was reduced quickly, and the CEE value fluctuated around 0.18. After processing according to Zheng [17], the CEE value fluctuated around 0.15, which was close to the SWF method. Lastly, after denoising by the IGSWF method, the deviation of the centroid extraction result was significantly reduced, and the CEE value fluctuated around 0.06. Based on the results, the CEE value after using the IGSWF algorithm was eight times smaller than the original value; six times smaller than that after traditional Gaussian filtering, box filtering, and mean filtering; and nearly three times smaller than that after the SWF and Zheng processing.

The comparison results show that the SWF and Zheng algorithm were superior to the traditional window-filtering algorithms. Although traditional Gaussian filtering could suppress the single-pixel noise, it could not improve the accuracy of the centroid extraction because it could not protect the shape and energy distribution of stars, which led to the deviation of the centroid extraction result. It should be noted that in this experiment, the proposed IGSWF algorithm achieved higher accuracy and better stability than the other compared methods. Moreover, the proposed IGSWF could improve the centroid extraction accuracy by several times and acquire a more precise attitude for the satellite.

Thirdly, the centroid extraction accuracy could improve the accuracy of star identification in the following process. Therefore, to verify the effect of the proposed IGSWF algorithm on the step of star identification, we compared the identification rate when we chose different algorithms, such as Gaussian filtering, SWF, and IGSWF. Typically, the triangular-based star-identification algorithm was selected.

In Table 2, it can be seen that the identification rate without a denoising algorithm was 85%. Due to the various errors of centroid extraction, the process of identification was instable. After Gaussian filtering and SWF processing, the identification rate was improved to 89.5% and 94.3%, respectively. After IGSWF processing, the identification rate was improved to 99.2%. So, it can be concluded that the proposed IGSWF algorithm was beneficial in improving the accuracy of star identification.

The results of the experiments listed above showed that the proposed IGSWF algorithm had a better ability than other algorithms in protecting the shape of stars and eliminating the single-pixel noise, and could improve the centroid extraction accuracy and the identification rate while maintaining high stability.

## 4. Discussion

In this paper, the IGSWF algorithm for star-image denoising based on the idea of edge protection was proposed. The proposed algorithm used four triangular Gaussian subtemplates, which is convenient for edge protection and engineering applications. In addition, based on the shape and energy characteristics of the star point, background, and image noise, a suitable calculation function $f_{min}\;$ for eliminating the single-pixel noise in a star image was introduced.

The proposed algorithm was verified by a simulation experiment, and the experimental results showed that the proposed IGSWF algorithm could maintain the edge characteristics of star points better than the traditional Gaussian filtering, box filtering, and mean filtering. It was also verified that IGSWF had higher precision than SWF in centroid extraction. Moreover, when processing the star image in-orbit, the IGSWF algorithm was adaptive to various environments of a satellite in orbit, and had an outstanding performance in single-pixel noise suppression.

Finally, through the application experiment, the accuracy and stability of the IGSWF algorithm were verified by comparing the CEE curves of centroid extraction for the original star image and the star image after Gaussian filtering, box filtering, mean filtering, Zheng processing, SWF processing, and IGSWF processing. The comparison results showed that, when the IGSWF algorithm was used, the accuracy (CEE value) of the centroid extraction was improved by nearly eight times compared to the original image, six times compared to the traditional window filtering, and three times compared to the SWF and Zheng. It also showed that the proposed IGSWF algorithm could improve the identification rate of the star sensor.

## 5. Conclusions

We concluded that the proposed IGSWF algorithm was better than the traditional window filter algorithms, Zheng, and SWF in preserving the shape of stars and eliminating the single-pixel noise, which was favorable to improving the precision of centroid extraction and the accuracy of identification rate.

In the future, the IGSWF algorithm will be applied to an on-orbit task for further verification and to improve the capability of the star sensor.

## Figures and Tables

**Table 1:** The center of mass.

Center of Mass	$\mathrm{Origin}\;(x_{o},\;y_{o})$	$\mathrm{IGSWF}\;(x_{n},\;y_{n})$	$\mathrm{SWF}\;({x^{\prime }}_{n},\;{y^{\prime }}_{n})$
Star 1	(36.883, 133.291)	(36.898, 133.258)	(36.910, 133.378)
Star 2	(446.025, 292.821)	(446.003, 292.860)	(446.000, 92.723)
Star 3	(608.824, 43.866)	(608.798, 43.882)	(608.766, 43.829)

**Table 2:** The identification rate.

Situations	Identification Rate (%)
Original	85.0%
Gaussian	89.5%
SWF	94.3%
IGSWF	99.2%

## Data Availability

Not applicable.
